# Prevalence and Global Distribution of Bacterial Species Associated with Acute Otitis Media in Children: Systematic Review and Meta-Analysis

**DOI:** 10.3390/antibiotics15050463

**Published:** 2026-05-03

**Authors:** Hye Ok Kim, Suhyeon Ha, Seung Hyung Lee, Yeon Ju Oh, Jae Min Lee, Youn-Jung Kim, Manish Kumar Singh, Sung Soo Kim, Jin Woo Choi, Seung Geun Yeo

**Affiliations:** 1 Department of Medicine, College of Medicine, Kyung Hee University, Seoul 02447, Republic of Korea; hyeokkim@khu.ac.kr (H.O.K.); lshl1220@khu.ac.kr (S.H.L.); 5duswn1203@khu.ac.kr (Y.J.O.); 2 Clinical Research Institute, Kyung Hee University Medical Center, Seoul 02447, Republic of Korea; yj129@khu.ac.kr; 3 Department of Pediatric Surgery, Asan Medical Center Children’s Hospital, Asan Medical Center, Seoul 05505, Republic of Korea; aa3246871@gmail.com; 4 Department of Otorhinolaryngology—Head and Neck Surgery, College of Medicine, Kyung Hee University Medical Center, Kyung Hee University, Seoul 02447, Republic of Korea; jmlee3042@khu.ac.kr; 5 College of Nursing Science, Kyung Hee University, Seoul 02447, Republic of Korea; 6 Department of Biochemistry and Molecular Biology, College of Medicine, Kyung Hee University, Seoul 02447, Republic of Korea; manishbiochem@gmail.com (M.K.S.); sgskim@khu.ac.kr (S.S.K.); 7 Department of Pharmacology, College of Pharmacy, Kyung Hee University, Seoul 02447, Republic of Korea; jinwoo.ch@khu.ac.kr

**Keywords:** acute otitis media, *Streptococcus pneumoniae*, *Haemophilus influenzae*, *Moraxella catarrhalis*, pediatric infection

## Abstract

**Background/Objectives:** Acute otitis media (AOM) remains a leading cause of pediatric morbidity and a primary indication for antibiotic prescription worldwide. Given the potential for serious complications and the evolving landscape of antimicrobial resistance, up-to-date epidemiological data on causative bacteria are essential. This study aimed to assess the global prevalence of major bacterial pathogens in pediatric AOM and evaluate variations across geographic regions and temporal periods (pre-2000 vs. post-2000). **Methods:** A systematic search of PubMed, Embase, and Web of Science (1980–2025) was conducted to identify studies reporting middle ear fluid culture results in children (0–18 years) with AOM. Following the Preferred Reporting Items for Systematic Reviews and Meta-Analyses (PRISMA) guidelines, 45 studies encompassing 16,305 AOM episodes were included. Data were synthesized from North America, Europe, the Middle East, Asia, Oceania, and Africa. Pooled prevalence estimates and 95% confidence intervals (CIs) were calculated using a random-effects model, and heterogeneity was assessed via the I^2^ statistic. **Results:** The overall pooled culture-positive rate was 66.6% (95% CI, 62.2–70.8%). Regional pooled estimates ranged from 56.1% in the Middle East (95% CI, 40.3–70.6%; underlying counts, 3776/10,652) to 77.5% in North America (95% CI, 68.2–84.7%; underlying counts, 1567/2125). *Streptococcus pneumoniae* was the most prevalent pathogen, with a pooled proportion of 29.0% (95% CI, 26.3–31.8%), followed by *Haemophilus influenzae* (22.3%; 95% CI, 19.3–25.6%) and *Moraxella catarrhalis* (4.6%; 95% CI, 3.4–6.1%). While *S. pneumoniae* remained the leading pathogen in most regions, *H. influenzae* showed marked geographic variability, peaking in the Middle East at 27.5% (95% CI, 17.0–41.2%; underlying counts, 2280/10,652) and reaching its lowest level in Asia at 13.5% (95% CI, 7.8–22.4%; underlying counts, 336/1854). The pooled culture-positive rate declined from 72.5% before 2000 (95% CI, 67.6–76.9%; underlying counts, 5769/8199) to 59.4% in 2000 and later (95% CI, 52.1–66.3%; underlying counts, 6661/15,707), although *S. pneumoniae* remained the predominant isolate in both periods. **Conclusions:** *S. pneumoniae* remains the primary bacterial driver of pediatric AOM globally. However, the observed geographic disparities and the temporal shift in pathogen prevalence following pneumococcal conjugate vaccine (PCV) introduction underscore the necessity for region-specific empirical antibiotic selection. These findings highlight the critical need for sustained microbiological surveillance to inform future vaccination and treatment strategies.

## 1. Introduction

Otitis media is an inflammatory condition of the middle ear that is especially common in young children and is classified as acute otitis media (AOM) or otitis media with effusion (OME) [1,2,3]. Two of three children who are under 3 years old experience at least one episode of AOM, and one of three children experiences three or more episodes [4,5]. The peak incidence of AOM is at age 6 to 12 months [6,7], but among different countries, the percentage of children with a first episode by age 1 year ranges from 19% to 62%, and the percentage with a first episode by age 3 years ranges from 50% to 84% [6,8,9].

AOM is characterized by a dysfunctional eustachian tube (ET), an organ that is relatively wider, shorter, and more horizontal in children, characteristics that facilitate the reflux of bacteria from the nasal cavity or nasopharynx into the middle ear. In addition, young children have immature cartilaginous support of the ET and of the muscles that control its opening [10,11]. These anatomical and physiological factors compromise ET function and are responsible for the higher incidence of AOM in infants and young children. The ET has a more mature structure and function in children aged 7 years, and AOM is less common in these older children. A functional ET normally prevents middle ear infections, but an immature or dysfunctional ET can allow pathogens to enter the middle ear. Most AOM-associated infections occur via the ET, although bacteria and viruses can also reach the middle ear through a perforated tympanic membrane or via hematogenous spread [10].

Most cases of AOM are attributed to *Streptococcus pneumoniae* followed by *Haemophilus influenzae* and *Moraxella catarrhalis*, and these three species together account for most culture-positive infections of middle ear fluid. *Group A β-hemolytic streptococci, α-hemolytic streptococci*, *Staphylococcus aureus*, and *Pseudomonas aeruginosa* are less common causes of AOM [12].

AOM is a major reason for antibiotic use in children, and this has led to the emergence of antimicrobial resistance in isolates from middle ear effusions. Depending on the region, approximately 8 to 34% of *S. pneumoniae* isolates, 25 to 30% of *H. influenzae* isolates, and about 80% of *M. catarrhalis* isolates have resistance to penicillin, and antibiotic resistance rates are steadily rising [13]. The percentage of β-lactamase-producing *H. influenzae* strains has also increased, and a recent study reported that 49.7% of *H. influenzae* strains isolated from upper respiratory tract infections, including AOM, acute tonsillitis, and acute sinusitis, were resistant to amoxicillin [14]. β-lactamase-nonproducing ampicillin-resistant H. influenzae (BLNAR) is also common, further complicating the treatment of these patients. In addition to antibiotic resistance, the widespread implementation of PCVs has altered the prevalence of AOM-associated pathogens. Importantly, nontypeable *H. influenzae* has become a more common cause of AOM in the post-PCV era. Thus, studies in many regions have reported that *H. influenzae* now rivals or even exceeds *S. pneumoniae* as the most common AOM pathogen in vaccinated populations [15]. These epidemiologic changes, coupled with differences in antibiotic resistance patterns among different pathogens, have complicated the management of AOM and underscore the critical importance of ongoing surveillance. Most previous studies of AOM pathogens have been confined to specific regions or time periods, highlighting the need for a broader perspective that integrates data from multiple countries and changes in these pathogens over time. A recent systematic review and meta-analysis of AOM reported that *S. pneumoniae*, *H. influenzae*, and *M. catarrhalis* were the three most common bacterial pathogens. However, this study did not specifically examine temporal changes or geographic variations in the prevalence of different species [16]. In the present study, we performed a meta-analysis of worldwide research on AOM pathogens to better understand global patterns and temporal changes in these pathogens.

## 2. Results

**Figure 1:**
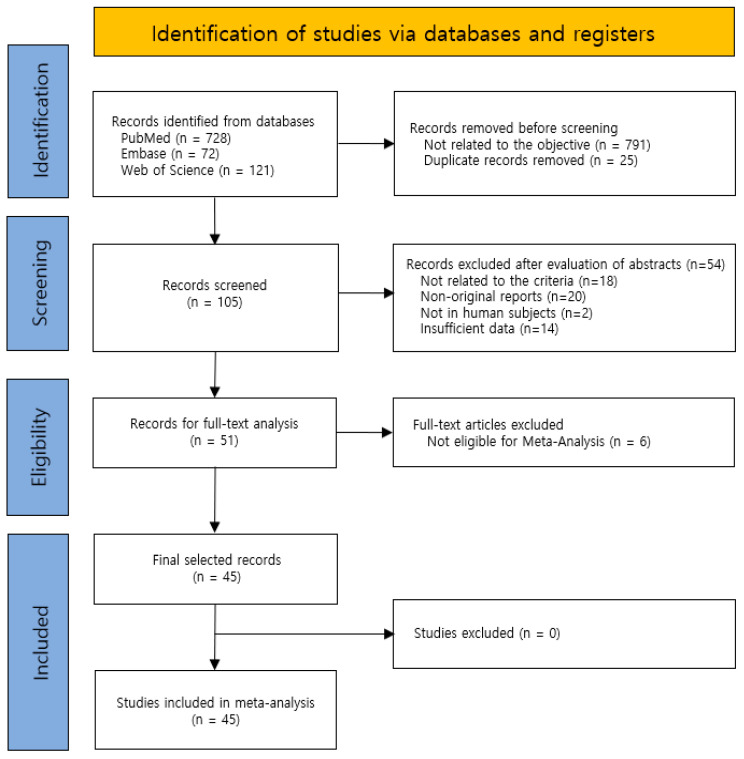
PRISMA flow diagram of the literature search strategy.

Our initial keyword search identified 921 articles and 51 of these studies [17,18,19,20,21,22,23,24,25,26,27,28,29,30,31,32,33,34,35,36,37,38,39,40,41,42,43,44,45,46,47,48,49,50,51,52,53,54,55,56,57,58,59,60,61,62,63,64,65,66,67] were eligible for full-text review (Figure 1, Supplementary Table S1). These 51 studies were from the Americas (21 from USA, Argentina, Brazil, Chile, Colombia, Costa Rica, Mexico, Venezuela), Europe (10 from Finland, France, Germany, Romania, Russia, Turkey), the Middle East (8 from Israel and Saudi Arabia), Asia (6 from China, Japan, Taiwan, Thailand), Oceania (1 from Australia), Africa (1 from Ethiopia), and multiple regions (4 from various countries, including USA, Costa Rica, Chile, Romania, Israel, Guatemala, Bulgaria, Peru, and Latvia). Six of these 51 articles did not meet the objectives of the meta-analysis. Thus, the final analysis included 45 articles: 21 from the Americas, 10 from Europe, 8 from the Middle East, and 6 from Asia. These articles contained data for 16,305 unique episodes of AOM (Supplementary Table S2).

The estimated percentage of positive cultures was 66.6% overall (95% CI: 0.622–0.708). The three most common species were *S. pneumoniae* (29.0%; 95% CI: 0.263–0.318), a Gram-positive diplococcus well-known to colonize upper respiratory tract mucosa; *H. influenzae* (22.3%; 95% CI: 0.193–0.256), a Gram-negative coccobacillus; and *M. catarrhalis* (4.6%; 95% CI: 0.034–0.061), a Gram-negative diplococcus (Table 1) [17,18,19,20,21,22,23,24,25,26,27,28,29,30,31,32,33,34,35,36,37,38,39,40,41,42,43,44,45,46,47,48,49,50,51,52,53,54,55,56,57,58,59,60,61,62,63,64,65,66,67].

We then compared the geographic distribution of species in North America (9 articles), South America (12 articles), Europe (10 articles), the Middle East (8 articles), and Asia (6 articles) (Table 1). For ‘Any species’, North America had the highest proportion of positive cultures (77.5%; 95% CI: 0.682–0.847) and the Middle East had the lowest proportion (56.1%; 95% CI: 0.403–0.706). *S. pneumoniae* was the most prevalent species in four of five regions. The pooled estimates for this species ranged from 25.2% (95% CI: 0.186–0.311) for the Middle East to 32.3% (95% CI: 0.285–0.364) for North America and 32.2% (95% CI: 0.231–0.429) for Asia. *H. influenzae* was the second most common pathogen overall. North America, South America, and Europe all had similar pooled *H. influenzae* detection rates around 23%. The Middle East had a slightly higher estimate at 27.5% (95% CI 0.170–0.412), whereas Asia’s estimate was markedly lower at 13.5% (95% CI 0.078–0.224). *M. catarrhalis* was the least common species in every region. North America had the highest pooled prevalence of this species (11.3%; 95% CI: 0.082–0.153), and this species was very rare in the Middle East (1.2%; 95% CI: 0.001–0.014).

We then compared the prevalence of different bacteria before and after the introduction of the PCV by using a meta-analysis to compare years up to and including 1999 (19 articles) and the year 2000 and later (24 articles). The results show that the overall culture-positive rate was lower in the post-2000 era (Table 2) [17,18,19,20,21,22,23,24,25,26,27,28,29,30,31,32,33,34,35,36,37,38,39,40,41,42,43,44,45,46,47,48,49,50,51,52,53,54,55,56,57,58,59,60,61,62,63,64,65,66,67]. Thus, for studies up to 1999, 72.5% (95% CI 0.676–0.769) of AOM cases were culture-positive; however, studies from 2000 and later had a culture-positive rate of 59.4% (95% CI 0.521–0.663). In addition, the positivity for each bacterial species was lower after 2000. Specifically, *S. pneumoniae* positivity decreased from 29.0% (95% CI 0.262–0.320) to 27.3% (95% CI 0.226–0.326); *H. influenzae* positivity decreased from 25.1% (95% CI 0.198–0.313) to 19.4% (0.159–0.233); and *M. catarrhalis* positivity decreased from 5.8% (0.058, 0.036–0.091) to 3.7% (0.037, 95% CI 0.023–0.058). Even though the positivity rates declined for all three species, *S. pneumoniae* was the most common species before and after 2000.

## 3. Discussion

In this first comprehensive global meta-analysis of AOM microbiology, we synthesized data from 45 studies (25,913 children) across multiple continents. Overall, approximately two-thirds of AOM cases were culture-positive for bacteria (~66%), indicating that bacteria account for the majority of episodes. *S. pneumoniae* was the most frequently isolated pathogen, responsible for about 29% of AOM cases, followed by *H. influenzae* (~22%) and *M. catarrhalis* (~5%) [68,69]. Together, these three organisms caused the vast majority of culture-confirmed AOM in children, reaffirming their dominant role in AOM etiology [66,67]. These findings confirm that *S. pneumoniae* remains the leading cause of AOM worldwide, with *H. influenzae* as a key secondary pathogen and *M. catarrhalis* a distant third in prevalence.

When broken down by region, North America had the highest proportion of AOM cases with any bacterial growth at 78%, whereas the Middle East had the lowest at 56%. Europe and Asia showed intermediate culture-positive rates of ~69% and ~59%, respectively.

When broken down by region, North America had the highest proportion of AOM cases with any bacterial growth at 78%, whereas the Middle East had the lowest at 56%. Europe and Asia showed intermediate culture-positive rates of ~69% and ~59%, respectively. Despite this variability, *S. pneumoniae* was the predominant AOM pathogen in all regions, contributing roughly one-quarter to one-third of cases (lowest ~25% in the Middle East, highest ~32% in North America and Asia; Table 1). *H. influenzae* was consistently the second most common isolate, but its relative prevalence varied widely—from only ~14% of AOM cases in Asia to ~28% in the Middle East. *M. catarrhalis* was the least frequent of the three major bacteria across all regions, peaking at ~11% of cases in North America and being rare in most other regions. Bacterial detection rates in AOM may be influenced by various factors, including the mode of infection, the timing of viral respiratory outbreaks (e.g., seasonal epidemics of common cold or influenza), patterns of antibiotic prescription and misuse, economic conditions, national healthcare policies, and the presence or absence of clinical guidelines for AOM. Accordingly, in the present study, we observed considerable variation in predominant pathogens across different continents. Because the spectrum of pathogens and the prevalence of antibiotic-resistant strains differ among countries, clinical practice guidelines for AOM also vary slightly from one country to another [7,70,71]. Our results underscore the importance of developing region-specific empiric antibiotic strategies in pediatric otolaryngology guidelines, so that treatment of AOM is aligned with the prevailing local pathogens and antimicrobial susceptibilities.

Regional treatment guidelines for pediatric acute otitis media (AOM) appear to be more concordant than divergent in their core empiric antibiotic recommendations. Across North American guidance, UK guidance, and available East Asian guidelines, amoxicillin remains the preferred first-line agent when antibiotics are indicated, whereas amoxicillin/clavulanate is generally reserved for children with recent amoxicillin exposure, concurrent purulent conjunctivitis, treatment failure, recurrent disease unresponsive to amoxicillin, or greater clinical severity. Korean guidance explicitly links this broader coverage to the increased likelihood of β-lactamase-producing *H. influenzae* and *M. catarrhalis*, and the Japanese 2018 update similarly emphasizes severity-based management informed by local bacteriology and antimicrobial susceptibility. Accordingly, the available regional guidelines differ less in their core antibiotic class than in the use of observation/watchful waiting, high-dose versus standard-dose amoxicillin, treatment duration, and thresholds for escalation. In this context, the predominance of *S. pneumoniae* in our pooled analysis supports the continued role of amoxicillin as the anchor first-line therapy in most settings. At the same time, broader β-lactamase coverage with amoxicillin/clavulanate is microbiologically plausible in scenarios where *H. influenzae* contributes more substantially or when clinical features suggest a higher probability of β-lactamase-producing pathogens. Thus, the regional differences observed in pathogen prevalence do not necessarily imply the need for fundamentally different empiric antibiotic classes, but rather support nuanced differences in when to broaden therapy and when observation may still be appropriate.

In 2000, the PCV was first licensed and introduced in the United States. This vaccine reduces the incidence of AOM and of invasive pneumococcal diseases, such as pneumonia, bacteremia, and meningitis. Accordingly, the U.S. Centers for Disease Control and Prevention (CDC) initially recommended PCV only for high-risk children (i.e., those with a cochlear implant or congenital inner ear malformation and an elevated risk of meningitis as a complication of AOM). However, since 2010, the CDC has recommended this vaccine for all infants and young children [72,73]. Other studies demonstrated that influenza vaccination was 30 to 55% effective in preventing AOM, and the U.S. CDC and the Korean immunization guidelines recommend annual influenza vaccination for all children older than 6 months [5,70,74]. The analysis also revealed clear temporal shifts in AOM pathogens corresponding to the introduction of PCVs in the early 2000s. In studies conducted before 2000, approximately 72.5% of AOM cases were culture-positive, whereas in 2000 and later, this fell to about 59.4%, indicating a substantial decline in bacterial isolation in the post-PCV era. The pooled prevalence of all three major bacteria (*S. pneumoniae*, *H. influenzae*, and *M. catarrhalis*) decreased after widespread PCV implementation. Notably, *S. pneumoniae* remained the leading cause of AOM in both the pre- and post-vaccine periods; however, in the Middle East, *H. influenzae* has recently overtaken *S. pneumoniae* as the top pathogen, suggesting vaccine-driven serotype replacement in that region. These findings highlight how vaccination programs can alter the AOM microbiologic landscape and reinforce the need for continued surveillance in the post-vaccine era. Despite vaccine-related declines in pneumococcal AOM, this disease remains highly prevalent and clinically significant worldwide—particularly among children under 5 years and in resource-limited regions where the burden of AOM complications is greatest. Ongoing monitoring of AOM pathogens is essential to inform immunization policies and update treatment guidelines as the pathogen spectrum evolves. Given that AOM is among the most common reasons for antibiotic prescriptions in children, strengthening antibiotic stewardship and aligning empiric therapy with current epidemiology are critical to optimize outcomes and curb resistance. Ultimately, our global findings provide a novel reference point for international clinical practice and public health policy. This evidence base can guide targeted vaccine programs and empiric treatment recommendations—especially in low-resource settings where local microbiology data may be sparse—with the goal of reducing the worldwide morbidity and antibiotic overuse associated with AOM.

The present study has certain limitations that must be acknowledged. First, the included studies varied widely in clinical context (geography, patient age, disease severity), introducing substantial heterogeneity. Consequently, true pathogen prevalence likely differs across populations and the pooled estimates should be interpreted with caution. Second, the data were unevenly distributed in space and time. Most studies originated from the Americas and Europe, whereas regions like Africa and Oceania were minimally represented, limiting generalizability to those areas. Combining data over broad time intervals may obscure year-to-year or vaccine-related shifts in pathogen prevalence, underscoring the need for more temporally granular data. In addition, much of the eligible microbiologic literature was relatively old and predated more recent vaccine transitions and newer molecular or genomic diagnostic approaches; accordingly, the pooled estimates may not fully represent the contemporary pathogen distribution in AOM. Third, an additional limitation of this review is that vaccination status and serotype-level microbiologic data could not be meta-analyzed. Most eligible studies did not report patient-level pneumococcal vaccination status or vaccination coverage in a standardized, extractable format, and serotype-specific results were available only in a limited subset of studies, often for selected pathogens and with heterogeneous denominators. Future primary studies should report vaccination exposure and serotype distributions systematically to enable more refined meta-analyses of vaccine-associated shifts in AOM microbiology. Fourth, statistical heterogeneity was high (I^2^ often >80%), indicating very high inter-study variability. Finally, our meta-analysis may be subject to publication bias, because many of the included studies were conducted in specialized settings, such as tertiary-care centers where tympanocentesis or myringotomy was used for sample collection. These settings likely enrolled patients with more severe AOM who underwent invasive sampling, which tends to yield a higher rate of culture positivity. Visual inspection of funnel plots by region suggested potential asymmetry, particularly for studies reporting higher rates of culture positivity, consistent with the possibility of publication bias.

## 4. Materials and Methods

### 4.1. Literature Search, Eligibility Criteria, and Study Selection

This systematic review and meta-analysis were conducted in accordance with the Preferred Reporting Items for Systematic Reviews and Meta-Analyses (PRISMA) guidelines (Figure 1 and Supplementary Table S3), and the protocol was registered in the International Prospective Register of Systematic Reviews (PROSPERO; CRD420251239168). PubMed, Embase, and Web of Science were searched for eligible studies published from 1 January 1980 to 31 December 2025 using the following database-specific Boolean search strings: PubMed, ((“acute otitis media”[tiab]) AND (bacteria[tiab] OR “Bacteriology”[MeSH Terms]) AND 1980:2025[dp]); Embase, ((“acute otitis media”:ti,ab) AND (bacteriology:ti,ab) AND [1980–2025]); and Web of Science, (TS = (“acute otitis media”) AND TS = (bacteriology) AND DY = (1980–2025)) (Supplementary Table S4).

Studies were eligible if they met all of the following criteria: (1) original human studies; (2) enrolled children or adolescents aged 0–18 years with acute otitis media; (3) reported bacterial findings from middle ear specimens obtained by tympanocentesis, myringotomy, or acute otorrhea clearly attributable to acute otitis media; and (4) provided extractable microbiological data, including at minimum the sample size and culture or pathogen results. Studies were excluded if they were non-original reports (e.g., reviews), did not involve human subjects, did not evaluate pediatric acute otitis media, used nasopharyngeal specimens only, were restricted to otorrhea from tympanostomy tubes, focused exclusively on recurrent acute otitis media or treatment failure, contained overlapping data (in which case the most recent or most comprehensive report was retained), or lacked sufficient data for analysis.

A total of 921 records were identified through database searching, including 728 from PubMed, 72 from Embase, and 121 from Web of Science. Before screening, 791 records were removed because they were not related to the review objective and 25 duplicate records were removed. The remaining 105 records underwent title and abstract screening. Of these, 54 records were excluded after abstract evaluation because they were not related to the eligibility criteria (n = 18), were non-original reports (n = 20), did not involve human subjects (n = 2), or contained insufficient data (n = 14). Subsequently, 51 full-text articles were assessed for eligibility. Six full-text articles were excluded because they were not eligible for quantitative synthesis. Ultimately, 45 studies met the eligibility criteria and were included in the meta-analysis, including 21 from the Americas, 10 from Europe, 8 from the Middle East, and 6 from Asia.

### 4.2. Data Extraction

Two reviewers (H.O.K. and S.H.H.) independently extracted data from each included study, and any discrepancies were resolved through discussion with a third reviewer (S.G.Y.). For each study, we extracted bibliographic and methodological information, including first author, publication year, country/region, study period, age range of participants, and specimen collection method. We also extracted clinical and microbiological variables, including pathogen detection method, and bacterial species identified.

For the quantitative synthesis, study-level numerators and denominators were extracted for each meta-analytic outcome. Specifically, we recorded the following: (1) the total number of middle ear fluid specimens analyzed, (2) the number of overall culture-positive specimens, and (3) the number of specimens positive for each bacterial pathogen of interest, including *S. pneumoniae*, *H. influenzae*, and *M. catarrhalis.* When more than one pathogen was identified in the same specimen, each pathogen-specific event was extracted separately for pathogen-specific analyses, whereas the overall culture-positive outcome counted the specimen only once. If acute otitis media data were reported as part of a broader study population, only AOM-specific data were extracted. Missing or unclear items were recorded as “not reported.”

### 4.3. Study Quality Assessment

Inclusion criteria were applied to ensure the quality of all included studies. Thus, only studies that satisfied all prespecified methodological criteria (e.g., clear case and outcome definitions and appropriate study design) were included. We assessed full texts for methodological quality and risk of bias using the Joanna Briggs Institute (JBI) Prevalence Critical Appraisal Tool [75]. Scoring of nine criteria, for responses including “yes” (one point), “no or unclear” (zero points) and “not applicable” (denominator reduced by one), produced a total score for each study. These are presented as percentages and categorised into three arbitrary levels: high risk of bias as 0–3 “Yes”, moderate risk 4–6 “Yes” and low risk 7–9 “Yes” [76]. The two researchers (H.O.K. and H.S.H.) evaluated the studies independently, and discrepancies were resolved through consensus and third-party researcher judgment (S.G.Y.). The results are presented in the risk of bias table (Supplementary Table S5).

### 4.4. Statistical Analysis

All extracted data were used for quantitative analysis. For each pathogen, the effect measure was defined as the prevalence (simple proportion) of patients with a positive culture, and the results are presented as pooled proportions with 95% confidence intervals (CIs). The data were tabulated using Microsoft Excel and analyzed using R version 4.5.1. A meta-analysis of the proportions was performed using the metaprop function from the meta package in R, and forest plots were generated using the forest function. For each outcome, pooled proportions and 95% CIs were computed using fixed-effect and random-effects models. Statistical heterogeneity was quantified by the I^2^ statistic. Because heterogeneity was extremely high (median I^2^ > 80%), the results were primarily interpreted using a random-effects model. We also performed a leave-one-out influence analysis using the metainf function in the R meta package. To explore potential sources of between-study heterogeneity, we conducted prespecified subgroup analyses according to region and study period (pre-2000 vs. 2000 and later).

## 5. Conclusions

In this meta-analysis of 45 studies comprising 16,305 pediatric AOM episodes, the overall pooled culture-positive rate was 66.6%. *S. pneumoniae* was the most prevalent pathogen worldwide (29.0%), followed by *H. influenzae* (22.3%) and *M. catarrhalis* (4.6%). Pathogen distribution showed clear regional variation, with the highest overall culture-positive rate in North America and the lowest in the Middle East, and with marked regional differences in the prevalence of *H. influenzae* and *M. catarrhalis*. Compared with studies conducted before 2000, studies from 2000 onward showed lower overall culture positivity (72.5% vs 59.4%) and lower pooled prevalence of all three major pathogens, although *S. pneumoniae* remained the leading isolate in both periods. These findings show that pediatric AOM microbiology is globally dominated by *S. pneumoniae* but varies substantially by region and has changed over time.

## Figures and Tables

**Table 1:** (**a**) Prevalence of the bacteria with AOM in different regions; (**b**) forest plots representing the prevalence of the bacteria with AOM in different regions [17,18,19,20,21,22,23,24,25,26,27,28,29,30,31,32,33,34,35,36,37,38,39,40,41,42,43,44,45,46,47,48,49,50,51,52,53,54,55,56,57,58,59,60,61,62,63,64,65,66,67].

(**a**)					
**Regions**	**Microbiology**				
	***Any Growth***		***S. pneumoniae***	***H. influenzae***	***M. catarrhalis***
North America	0.775 (0.682, 0.847)		0.323 (0.285, 0.364)	0.233 (0.197, 0.273)	0.113 (0.082, 0.153)
South America	0.657 (0.592, 0.717)		0.280 (0.236, 0.329)	0.233 (0.178, 0.298)	0.026 (0.016, 0.042)
Europe	0.689 (0.574, 0.784)		0.267 (0.181, 0.375)	0.233 (0.197, 0.273)	0.072 (0.044, 0.115)
Middle East	0.561 (0.403, 0.706)		0.252 (0.186, 0.311)	0.275 (0.170, 0.412)	0.012 (0.001, 0.014)
Asia	0.586 (0.469, 0.694)		0.322 (0.231, 0.429)	0.135 (0.078, 0.224)	0.031 (0.011, 0.080)
Overall	0.666 (0.622, 0.708)		0.290 (0.263, 0.318)	0.223 (0.193, 0.256)	0.046 (0.034, 0.061)
(**b**)					
**Microbiology**		**North America**		**South America**	**Europe**
***Any growth***		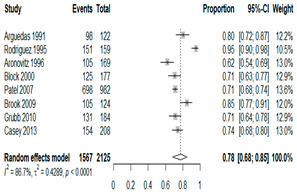		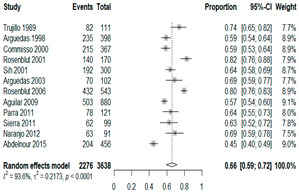	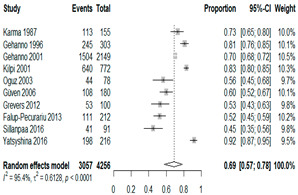
		**Middle East**		**Asia**	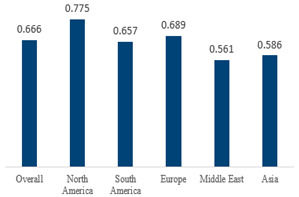
		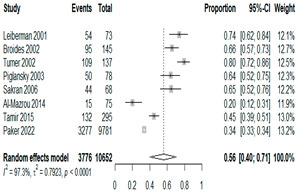		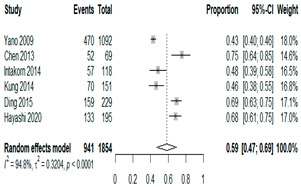	
***S. pneumoniae***		** North America **		**South America**	**Europe**
		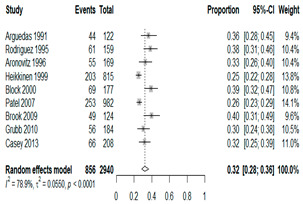		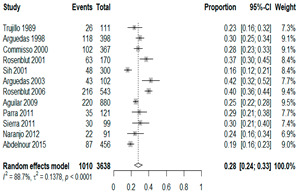	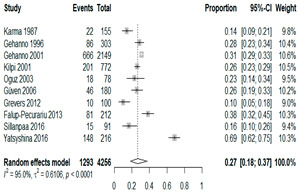
		**Middle East**		**Asia**	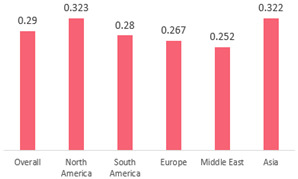
		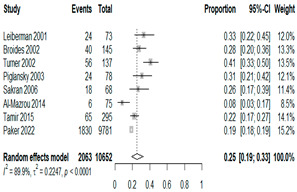		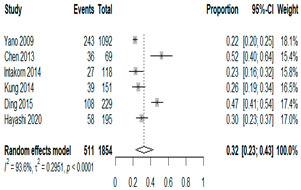	
***H. influenzae***		** North America **		**South America**	**Europe**
		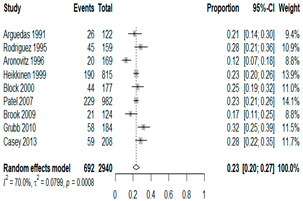		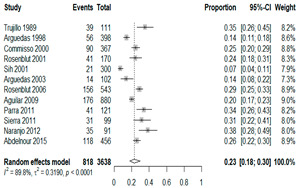	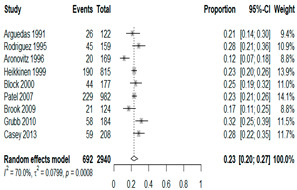
		**Middle East**		**Asia**	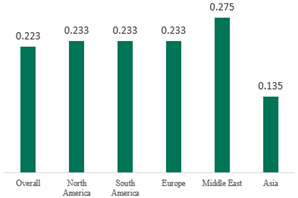
		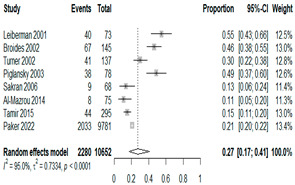		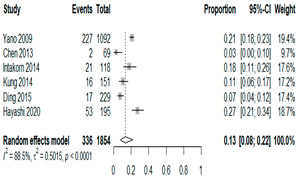	

**Table 2:** (**a**) Prevalence of the bacteria with AOM during different periods; (**b**) forest plots representing the prevalence of the bacteria with AOM during different periods [17,18,19,20,21,22,23,24,25,26,27,28,29,30,31,32,33,34,35,36,37,38,39,40,41,42,43,44,45,46,47,48,49,50,51,52,53,54,55,56,57,58,59,60,61,62,63,64,65,66,67].

(**a**)			
**Bacteriology**	**Overall**	**Pre-2000**	**Post-2000**
*Any growth*	0.663 (0.618, 0.705)	0.725 (0.676, 0.769)	0.594 (0.521, 0.663)
*S. pneumoniae*	0.288 (0.261, 0.317)	0.290 (0.262, 0.320)	0.273 (0.226, 0.326)
*H. influenzae*	0.223 (0.192, 0.257)	0.251 (0.198, 0.313)	0.194 (0.159, 0.233)
*M. catarrhalis*	0.045 (0.034, 0.061)	0.058 (0.036, 0.091)	0.037 (0.023, 0.058)
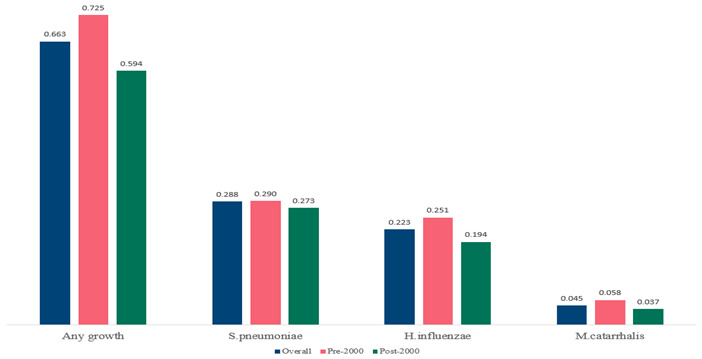			
(**b**)			
**Microbiology**	**Pre-2000**	**Post-2000**	
*Any growth*	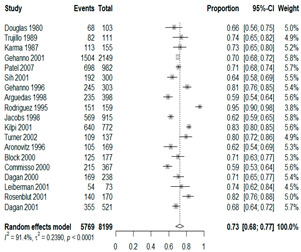	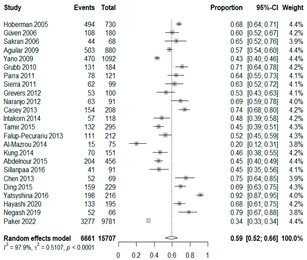	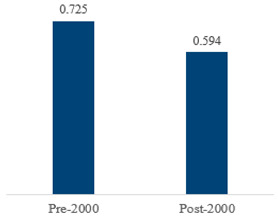
*S. pneumoniae*	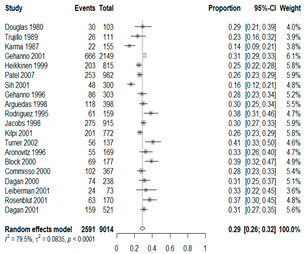	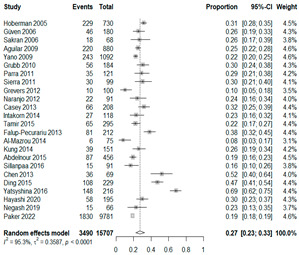	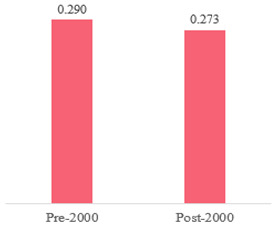
**Microbiology**	**Pre-2000**	**Post-2000**	
*H. influenzae*	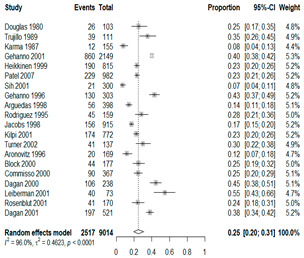	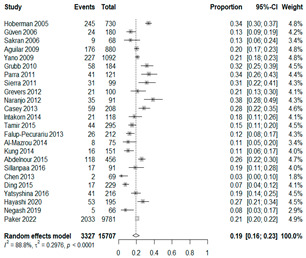	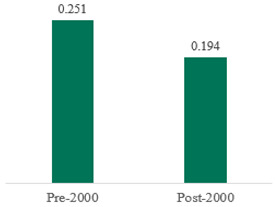
*M. catarrhalis*	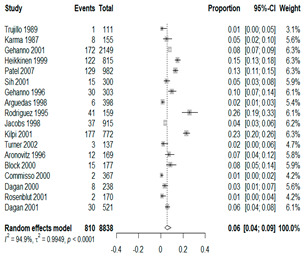	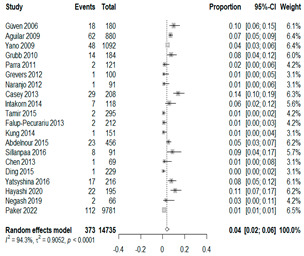	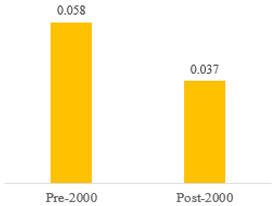

## Data Availability

The data used to support the findings of this study are included within the article and Supplementary files.

## Supplementary Materials

The following supporting information can be downloaded at: https://www.mdpi.com/article/10.3390/antibiotics15050463/s1, Table S1: Characteristics of Included Studies; Table S2: Microbiology culture results from 16,305 bacteria isolate with AOM; Table S3: PRISMA 2020 checklist; Table S4: Search strategy from databases; Table S5: Risk of bias for all included studies.
