# Supplementary material for: Prevalence and Global Distribution of Bacterial Species Associated with Acute Otitis Media in Children: Systematic Review and Meta-Analysis
Source: Antibiotics (Basel). 2026 May 3;15(5):463. doi: 10.3390/antibiotics15050463 (PMC13203586; doi:10.3390/antibiotics15050463)
Supplement: Supplementary file 1 [file antibiotics-15-00463-s001.zip › antibiotics-4252269-supplementary.pdf]

Supplementary Table S1. Characteristics of Included Studies

Country /Region	Year study commenced	Year study ended	Lower age limit of participants (months)	Upper age limit of participants (months)	AOM cases /episodes	References
**North America**						
USA	ND	ND	ND	ND	122	Arguedas et al. (1991)
USA	1994	1995	12	72	159	Rodriguez et al. (1995)
USA	1996	1996	24	180	169	Aronovitz (1996)
USA	1989	1993	2	84	815	Heikkinen et al. (1999)
USA	1996	1996	6	144	177	Block et al. (2000)
USA	1989	1998	2	84	982	Patel et al. (2007)
USA	1998	2006	5	144	124	Brook and Gober (2009)
USA	2005	2009	ND	ND	184	Grubb and Spaugh (2010)
USA	2008	2010	6	36	208	Casey et al. (2013)
**South America**						
Argentina	1996	1997	1	24	367	Commisso et al. (2000)
Brazil	1990	1995	2	60	300	Sih (2001)
Chile	1998	1999	3	ND	170	Rosenblut et al. (2001)
Chile	1998	2002	3	108	543	Rosenblut et al. (2006)
Colombia	1979	1985	0.1	132	111	Trujillo et al. (1989)
Colombia	2008	2009	3	60	99	Sierra et al. (2011)
Costa Rica	1992	1997	4	144	398	Arguedas et al. (1998)
Costa Rica	1999	2001	4	123	102	Arguedas et al. (2003)
Costa Rica	2002	2007	2	92	880	Aguilar et al. (2009)
Costa Rica	2010	2012	3	59	456	Abdelnour et al. (2015)
Mexico	2008	2009	3	60	121	Parra et al. (2011)
Venezuela	2008	2009	0	60	91	Naranjo et al. (2012)
**Europe**						
Finland	1980	1985	0	2	155	Karma et al. (1987)
Finland	1994	1995	2	24	772	Kilpi et al. (2001)
Finland	2010	2011	6	39	91	Sillanpaa et al. (2016)
France	1992	1993	0	71	303	Gehanno et al. (1996)
France	1987	1997	3	36	2149	Gehanno et al. (2001)
Germany	2008	2010	3	60	100	Grevers et al. (2012)
Romania	2009	2011	0	59	212	Falup-Pecurariu et al. (2013)
Russia	2011	2013	0	59	216	Yatsyshina et al. (2016)

Turkey	2002	2004	6	144	180	Güven et al. (2006)
Turkey	1998	2000	6	144	78	Oguz et al. (2003)
**Middle East**						
Israel	1998	1999	3	32	73	Leiberman et al. (2001)
Israel	1998	2000	3	36	145	Broides et al. (2002)
Israel	1995	1999	0	2	137	Turner et al. (2002)
Israel	1999	2001	3	36	78	Piglansky et al. (2003)
Israel	2002	2004	0	2	68	Sakran et al. (2006)
Israel	2008	2013	0	72	295	Tamir et al. (2015)
Israel	2002	2019	0	60	9781	Paker et al. (2022)
Saudi Arabia	2009	2011	3	60	75	Al-Mazrou et al. (2014)
**Asia**						
China [40]	2011	2013	0	216	229	Ding et al. (2015)
Japan	2002	2004	0	120	1092	Yano et al. (2009)
Japan	2011	2012	0	180-	195	Hayashi et al. (2020)
Taiwan	2011	2012	0	215	69	Chen et al. (2013)
Taiwan	2009	2011	4	213	151	Kung et al. (2014)
Thailand	2008	2009	5	59	118	Intakorn et al. (2014)
***Oceania**						
Australia	1977	1978	4	168	103	Douglas et al. (1980)
***Africa**						
Ethiopia	2016	2017	0	180	66	Negash et al. (2019)
***Etc.**						
International	1994	1995	3	60	915	Jacobs et al. (1998)
International	1997	1998	3.9	106	238	Dagan et al. (2000)
International	1999	1999	3	48	521	Dagan et al. (2001)
International	2001	2002	6	30	730	Hoberman et al. (2005)

*Study not included in the meta-analysis.

Supplementary Table S2. Microbiology culture results from 16,305 bacteria isolate with AOM

Microbiology	No. of Isolates, n (%)
Streptococcus pneumoniae	6,515 (40)
Haemophilus influenzae	6,177 (38)
Moraxella catarrhalis	1,234 (8)
Streptococcus pyogenes	1,223 (8)
Staphylococcus aureus	401 (2)
Coagulase-negative staphylococci	387 (2)
Pseudomonas aeruginosa	126 (1)
[a]*Others*	242 (1)
Total	16,305 (100)

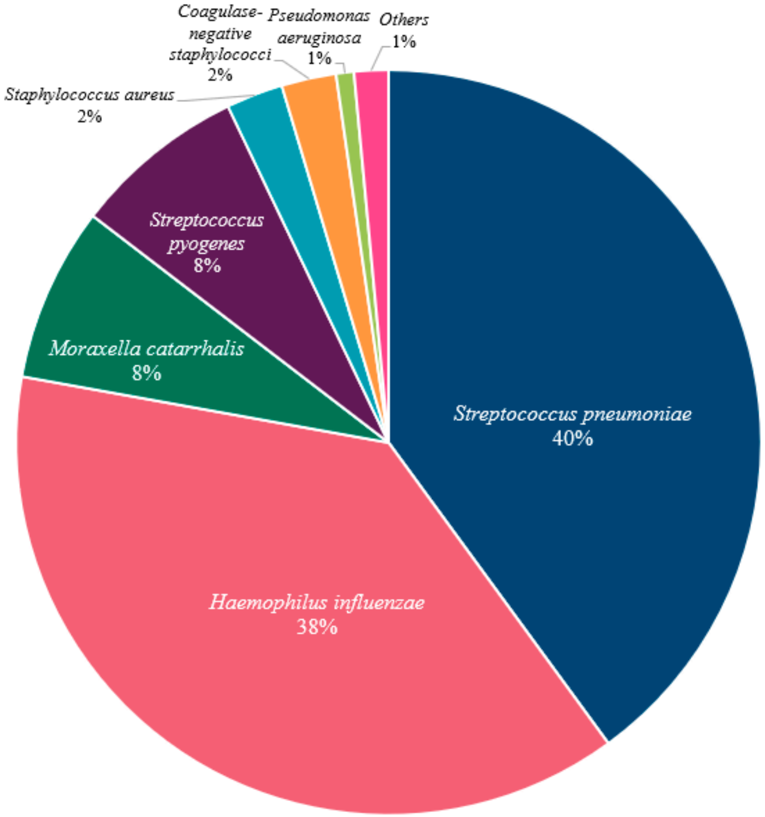

[a]Includes: *Proteus spp., Klebsiella spp., Streptococcus viridans, Enterococcus spp., Corynebacterium spp., Acinetobacter baumannii, Neisseria spp., Bacillus spp., Streptococcus mitis, Micrococcus spp., Haemophilus parainfluenzae* etc.

Supplementary Table S3. PRISMA 2020 Checklist

Section and Topic	Item #	Checklist item	Location where item is reported
**TITLE**			
Title	1	Identify the report as a systematic review.	Page 1
**ABSTRACT**			
Abstract	2	See the PRISMA 2020 for Abstracts checklist.	Page 3-4
**INTRODUCTION**			
Rationale	3	Describe the rationale for the review in the context of existing knowledge.	Page 4-6
Objectives	4	Provide an explicit statement of the objective(s) or question(s) the review addresses.	Page 6
**METHODS**			
Eligibility criteria	5	Specify the inclusion and exclusion criteria for the review and how studies were grouped for the syntheses.	Page 6-7
Information sources	6	Specify all databases, registers, websites, organisations, reference lists and other sources searched or consulted to identify studies. Specify the date when each source was last searched or consulted.	Page 6-7
Search strategy	7	Present the full search strategies for all databases, registers and websites, including any filters and limits used.	Page 6-7
Selection process	8	Specify the methods used to decide whether a study met the inclusion criteria of the review, including how many reviewers screened each record and each report retrieved, whether they worked independently, and if applicable, details of automation tools used in the process.	Page 7
Data collection process	9	Specify the methods used to collect data from reports, including how many reviewers collected data from each report, whether they worked independently, any processes for obtaining or confirming data from study investigators, and if applicable, details of automation tools used in the process.	Page 8
Data items	10a	List and define all outcomes for which data were sought. Specify whether all results that were compatible with each outcome domain in each study were sought (e.g. for all measures, time points, analyses), and if not, the methods used to decide which results to collect.	Page 8
	10b	List and define all other variables for which data were sought (e.g. participant and intervention characteristics, funding sources). Describe any assumptions made about any missing or unclear information.	Page 8
Study risk of bias assessment	11	Specify the methods used to assess risk of bias in the included studies, including details of the tool(s) used, how many reviewers assessed each study and whether they worked independently, and if applicable, details of automation tools used in the process.	Page 8
Effect measures	12	Specify for each outcome the effect measure(s) (e.g. risk ratio, mean difference) used in the synthesis or presentation of results.	Page 9
Synthesis methods	13a	Describe the processes used to decide which studies were eligible for each synthesis (e.g. tabulating the study intervention characteristics and comparing against the planned groups for each synthesis (item #5)).	Page 9

Section and Topic	Item #	Checklist item	Location where item is reported
	13b	Describe any methods required to prepare the data for presentation or synthesis, such as handling of missing summary statistics, or data conversions.	Page 8
	13c	Describe any methods used to tabulate or visually display results of individual studies and syntheses.	Page 9
	13d	Describe any methods used to synthesize results and provide a rationale for the choice(s). If meta-analysis was performed, describe the model(s), method(s) to identify the presence and extent of statistical heterogeneity, and software package(s) used.	Page 9
	13e	Describe any methods used to explore possible causes of heterogeneity among study results (e.g. subgroup analysis, meta-regression).	Page 9
	13f	Describe any sensitivity analyses conducted to assess robustness of the synthesized results.	Page 9
Reporting bias assessment	14	Describe any methods used to assess risk of bias due to missing results in a synthesis (arising from reporting biases).	Page 8
Certainty assessment	15	Describe any methods used to assess certainty (or confidence) in the body of evidence for an outcome.	Page 8-9
**RESULTS**			
Study selection	16a	Describe the results of the search and selection process, from the number of records identified in the search to the number of studies included in the review, ideally using a flow diagram.	Page 9-10, Figure 1
	16b	Cite studies that might appear to meet the inclusion criteria, but which were excluded, and explain why they were excluded.	Page 7
Study characteristics	17	Cite each included study and present its characteristics.	Page 8, Table 2
Risk of bias in studies	18	Present assessments of risk of bias for each included study.	Page 7
Results of individual studies	19	For all outcomes, present, for each study: (a) summary statistics for each group (where appropriate) and (b) an effect estimate and its precision (e.g. confidence/credible interval), ideally using structured tables or plots.	Page 9-11, Table 2,3
Results of syntheses	20a	For each synthesis, briefly summarise the characteristics and risk of bias among contributing studies.	Page 8
	20b	Present results of all statistical syntheses conducted. If meta-analysis was done, present for each the summary estimate and its precision (e.g. confidence/credible interval) and measures of statistical heterogeneity. If comparing groups, describe the direction of the effect.	Table 2,3
	20c	Present results of all investigations of possible causes of heterogeneity among study results.	Table 2,3
	20d	Present results of all sensitivity analyses conducted to assess the robustness of the synthesized results.	Page 8
Reporting biases	21	Present assessments of risk of bias due to missing results (arising from reporting biases) for each synthesis assessed.	Page 8
Certainty of evidence	22	Present assessments of certainty (or confidence) in the body of evidence for each outcome assessed.	Page 9-11,

Section and Topic	Item #	Checklist item	Location where item is reported
			Table 2,3
**DISCUSSION**			
Discussion	23a	Provide a general interpretation of the results in the context of other evidence.	Page 11-13
	23b	Discuss any limitations of the evidence included in the review.	Page 14-15
	23c	Discuss any limitations of the review processes used.	Page 14-15
	23d	Discuss implications of the results for practice, policy, and future research.	Page 14-15
**OTHER INFORMATION**			
Registration and protocol	24a	Provide registration information for the review, including register name and registration number, or state that the review was not registered.	Page 17
	24b	Indicate where the review protocol can be accessed, or state that a protocol was not prepared.	Page 17
	24c	Describe and explain any amendments to information provided at registration or in the protocol.	Page 17
Support	25	Describe sources of financial or non-financial support for the review, and the role of the funders or sponsors in the review.	Page 16
Competing interests	26	Declare any competing interests of review authors.	Page 16
Availability of data, code and other materials	27	Report which of the following are publicly available and where they can be found: template data collection forms; data extracted from included studies; data used for all analyses; analytic code; any other materials used in the review.	Page 17

Supplementary Table S4. Search strategy from databases

1. PubMed
(“acute otitis media”[tiab]) AND (bacteria[tiab] OR “Bacteriology”[MeSH Terms]) AND 1980:2025[dp]
2. Embase
(“acute otitis media”:ti,ab) AND (bacteriology:ti,ab) AND [1980-2025]
3. Web of Science
TS=(“acute otitis media”) AND TS=(bacteriology) AND DY=(1980-2025)

Supplementary Table S5. Risk of bias for all included studies (JBI Tool)

Study Author, (Year)	Q1	Q2	Q3	Q4	Q5	Q6	Q7	Q8	Q9	Yes (n)	Yes (%)	Risk of Bias
**Arguedas et al. (1991)**	No	Yes	No	Yes	Yes	Yes	Yes	Yes	Yes	7	78%	Low
**Rodriguez et al. (1995)**	No	Yes	No	Yes	Yes	Yes	Yes	Yes	Yes	7	78%	Low
**Aronovitz (1996)**	No	Yes	No	Yes	Yes	Yes	Yes	Yes	Yes	7	78%	Low
**Heikkinen et al. (1999)**	No	Yes	Yes	Yes	Yes	Yes	Yes	Yes	Yes	8	89%	Low
**Block et al. (2000)**	No	Yes	No	Yes	No	Yes	Yes	Yes	Yes	6	67%	Moderate
**Patel et al. (2007)**	No	No	Yes	Yes	No	Yes	Yes	Yes	Yes	6	67%	Moderate
**Brook and Gober (2009)**	No	Yes	No	Yes	Yes	Yes	Yes	Yes	Yes	7	78%	Low
**Grubb and Spaugh (2010)**	No	Yes	No	Yes	Yes	Yes	Yes	Yes	Yes	7	78%	Low
**Casey et al. (2013)**	No	Yes	Yes	Yes	Yes	Yes	Yes	Yes	Yes	8	89%	Low
**Commisso et al. (2000)**	No	Yes	Yes	Yes	Yes	Yes	Yes	Yes	Yes	8	89%	Low
**Sih (2001)**	No	Yes	Yes	Yes	Yes	Yes	Yes	Yes	Yes	8	89%	Low
**Rosenblut et al. (2001)**	No	Yes	No	Yes	Yes	Yes	Yes	Yes	Yes	7	78%	Low
**Rosenblut et al. (2006)**	No	Yes	Yes	Yes	Yes	Yes	Yes	Yes	Yes	8	89%	Low
**Trujillo et al. (1989)**	No	Yes	No	Yes	Yes	Yes	Yes	Yes	Yes	7	78%	Low
**Sierra et al. (2011)**	No	Yes	No	Yes	Yes	Yes	Yes	Yes	Yes	7	78%	Low
**Arguedas et al. (1998)**	No	Yes	Yes	Yes	Yes	Yes	Yes	Yes	Yes	8	89%	Low
**Arguedas et al. (2003)**	No	Yes	No	Yes	Yes	Yes	Yes	Yes	Yes	7	78%	Low

**Aguilar et al. (2009)**	No	Yes	Yes	Yes	Yes	Yes	Yes	Yes	Yes	8	89%	Low
**Abdelnour et al. (2015)**	No	Yes	Yes	Yes	Yes	Yes	Yes	Yes	Yes	8	89%	Low
**Parra et al. (2011)**	Yes	Yes	No	Yes	Yes	Yes	Yes	Yes	Yes	7	78%	Low
**Naranjo et al. (2012)**	No	Yes	No	Yes	Yes	Yes	Yes	Yes	Yes	7	78%	Low
**Karma et al. (1987)**	No	Yes	No	Yes	Yes	Yes	Yes	Yes	Yes	7	78%	Low
**Kilpi et al. (2001)**	Yes	Yes	Yes	Yes	Yes	Yes	Yes	Yes	Yes	9	100%	Low
**Sillanpaa et al. (2016)**	No	Yes	No	Yes	No	Yes	Yes	Yes	Yes	6	67%	Moderate
**Gehanno et al. (1996)**	No	Yes	Yes	Yes	Yes	Yes	Yes	Yes	Yes	8	89%	Low
**Gehanno et al. (2001)**	No	Yes	Yes	Yes	Yes	Yes	Yes	Yes	Yes	8	89%	Low
**Grevers et al. (2012)**	No	Yes	No	Yes	Yes	Yes	Yes	Yes	Yes	7	78%	Low
**Falup-Pecurariu et al. (2013)**	No	Yes	Yes	Yes	Yes	Yes	Yes	Yes	Yes	8	89%	Low
**Yatsyshina et al. (2016)**	No	Yes	Yes	Yes	Yes	Yes	Yes	Yes	Yes	8	89%	Low
**Güven et al. (2006)**	No	Yes	No	Yes	Yes	Yes	Yes	Yes	Yes	7	78%	Low
**Oguz et al. (2003)**	No	Yes	No	Yes	Yes	Yes	Yes	Yes	Yes	7	78%	Low
**Leiberman et al. (2001)**	No	Yes	No	Yes	No	Yes	Yes	Yes	Yes	6	67%	Moderate
**Broides et al. (2002)**	No	No	No	Yes	No	Yes	Yes	Yes	Yes	5	56%	Moderate
**Turner et al. (2002)**	No	Yes	No	Yes	Yes	Yes	Yes	Yes	Yes	7	78%	Low
**Piglansky et al. (2003)**	No	Yes	No	Yes	No	Yes	Yes	Yes	Yes	6	67%	Moderate
**Sakran et al. (2006)**	No	Yes	No	Yes	Yes	Yes	Yes	Yes	Yes	7	78%	Low

**Tamir et al. (2015)**	No	Yes	Yes	Yes	Yes	Yes	Yes	Yes	Yes	8	89%	Low
**Paker et al. (2022)**	No	Yes	Yes	Yes	Yes	Yes	Yes	Yes	Yes	8	89%	Low
**Al-Mazrou et al. (2014)**	No	Yes	No	Yes	Yes	Yes	Yes	Yes	Yes	7	78%	Low
**Ding et al. (2015)**	No	Yes	Yes	Yes	Yes	Yes	Yes	Yes	Yes	8	89%	Low
**Yano et al. (2009)**	No	Yes	Yes	Yes	Yes	Yes	Yes	Yes	Yes	8	89%	Low
**Hayashi et al. (2020)**	No	No	No	Yes	No	Yes	Yes	Yes	Yes	5	56%	Moderate
**Chen et al. (2013)**	No	Yes	No	Yes	No	Yes	Yes	Yes	Yes	5	56%	Moderate
**Kung et al. (2014)**	No	Yes	No	Yes	Yes	Yes	Yes	Yes	Yes	7	78%	Low
**Intakorn et al. (2014)**	No	Yes	No	Yes	Yes	Yes	Yes	Yes	Yes	7	78%	Low
**Douglas et al. (1980)**	No	No	No	Yes	No	Yes	Yes	Yes	Yes	5	56%	Moderate
**Negash et al. (2019)**	No	Yes	No	Yes	Yes	Yes	Yes	Yes	Yes	7	78%	Low
**Jacobs et al. (1998)**	No	Yes	Yes	Yes	Yes	Yes	Yes	Yes	Yes	8	89%	Low
**Dagan et al. (2000)**	No	Yes	Yes	Yes	No	Yes	Yes	Yes	Yes	7	78%	Low
**Dagan et al. (2001)**	No	Yes	Yes	Yes	Yes	Yes	Yes	Yes	Yes	8	89%	Low
**Hoberman et al. (2005)**	No	Yes	Yes	Yes	Yes	Yes	Yes	Yes	Yes	8	89%	Low
